# A Conserved Non-Reproductive GnRH System in Chordates

**DOI:** 10.1371/journal.pone.0041955

**Published:** 2012-07-27

**Authors:** Takehiro G. Kusakabe, Tsubasa Sakai, Masato Aoyama, Yuka Kitajima, Yuki Miyamoto, Toru Takigawa, Yutaka Daido, Kentaro Fujiwara, Yasuko Terashima, Yoko Sugiuchi, Giorgio Matassi, Hitoshi Yagisawa, Min Kyun Park, Honoo Satake, Motoyuki Tsuda

**Affiliations:** 1 Department of Biology, Faculty of Science and Engineering, Konan University, Kobe, Japan; 2 Graduate School of Life Science, University of Hyogo, Kamigori, Hyogo, Japan; 3 Division of Biomolecular Research, Suntory Institute for Bioorganic Research, Shimamoto, Osaka, Japan; 4 Department of Agriculture and Environmental Sciences, University of Udine, Udine, Italy; 5 Department of Biological Sciences, Graduate School of Science, the University of Tokyo, Tokyo, Japan; 6 Kagawa School of Pharmaceutical Sciences, Tokushima Bunri University, Sanuki, Kagawa, Japan; Laboratoire Arago, France

## Abstract

Gonadotropin-releasing hormone (GnRH) is a neuroendocrine peptide that plays a central role in the vertebrate hypothalamo-pituitary axis. The roles of GnRH in the control of vertebrate reproductive functions have been established, while its non-reproductive function has been suggested but less well understood. Here we show that the tunicate *Ciona intestinalis* has in its non-reproductive larval stage a prominent GnRH system spanning the entire length of the nervous system. Tunicate GnRH receptors are phylogenetically closest to vertebrate GnRH receptors, yet functional analysis of the receptors revealed that these simple chordates have evolved a unique GnRH system with multiple ligands and receptor heterodimerization enabling complex regulation. One of the *gnrh* genes is conspicuously expressed in the motor ganglion and nerve cord, which are homologous structures to the hindbrain and spinal cord of vertebrates. Correspondingly, GnRH receptor genes were found to be expressed in the tail muscle and notochord of embryos, both of which are phylotypic axial structures along the nerve cord. Our findings suggest a novel non-reproductive role of GnRH in tunicates. Furthermore, we present evidence that GnRH-producing cells are present in the hindbrain and spinal cord of the medaka, *Oryzias latipes*, thereby suggesting the deep evolutionary origin of a non-reproductive GnRH system in chordates.

## Introduction

Gonadotropin-releasing hormone (GnRH) plays a pivotal role in controlling reproductive functions in vertebrates. Three major GnRH systems have been characterized in vertebrates: the conventional hypophysiotropic GnRH (GnRH1) system and two extrahypothalamic GnRH (GnRH2 and GnRH3) systems [1]–[4]. The hypothalamic GnRH1 regulates the reproductive endocrine system by facilitating the release of gonadotropins from the pituitary. The cell bodies of the GnRH2 and GnRH3 systems are localized in the midbrain and the terminal nerves, respectively. Whether hypophysiotropic or extrahypothalamic, most functions of the GnRH systems have been implicated in the control of reproductive activities, such as gonad development, gonad function, and reproductive behaviors [2], [3]. Non-reproductive roles of GnRH have been suggested by the broad distribution of GnRH receptors (GnRHRs) in the central nervous system [5]–[8] as well as by projection of GnRH neurons to wide areas in the brain [3], [9]. Furthermore, the presence of GnRH in early embryos and experimental manipulation of GnRH activity suggest developmental roles of GnRH in vertebrate embryos [10]–[14]. Compared to its reproductive roles, however, the non-reproductive roles of GnRH are less well understood.

Tunicates are the sister group of vertebrates [15], [16]. It has been suggested that GnRH is involved in reproductive control in sessile tunicates, namely ascidians [17]–[19]. Despite their simplicity in anatomical organization and reproductive mode, GnRHs and their receptors exhibit a rich diversity in tunicates; six GnRH peptides and four receptors are encoded by the genome of the ascidian *Ciona intestinalis* [19]–[21]. This molecular complexity of the GnRH system leads us to hypothesize a non-reproductive role of GnRH in ascidians as well. Such a non-reproductive role of GnRH has been suggested by the presence of transcripts encoding GnRH in *Ciona* embryos and larvae [19]. GnRH-like immunoreactivity in adult sensory neurons of some species also suggests non-reproductive roles of GnRH in ascidians [22], [23].

The roles and localization of GnRHs have been investigated in adult tunicates, which exhibit an extremely modified form as chordates, while little is known about GnRH systems in the larvae, which exhibit a well-conserved chordate body plan. Here we show a novel non-reproductive feature of the GnRH system in *C. intestinalis* larvae. Our results suggest that tunicates evolved a sophisticated GnRH system with specific interactions between multiple ligands and receptors. The GnRH genes are conspicuously expressed in the central nervous system (CNS) through the entire antero-posterior body axis. Correspondingly, the GnRH receptor genes are specifically expressed in the phylotypic axial structures along the CNS. We further present evidence for localization of cells producing GnRH in the hindbrain and spinal cord of vertebrates. Our findings demonstrate that the GnRH system is deeply associated with the chordate-specific characteristics and suggest the evolutionary conservation of a non-reproductive GnRH system between tunicates and vertebrates.

## Results

### Prominent Expression of *gnrh* Genes in the *Ciona intestinalis* Larva

**Figure 1:**
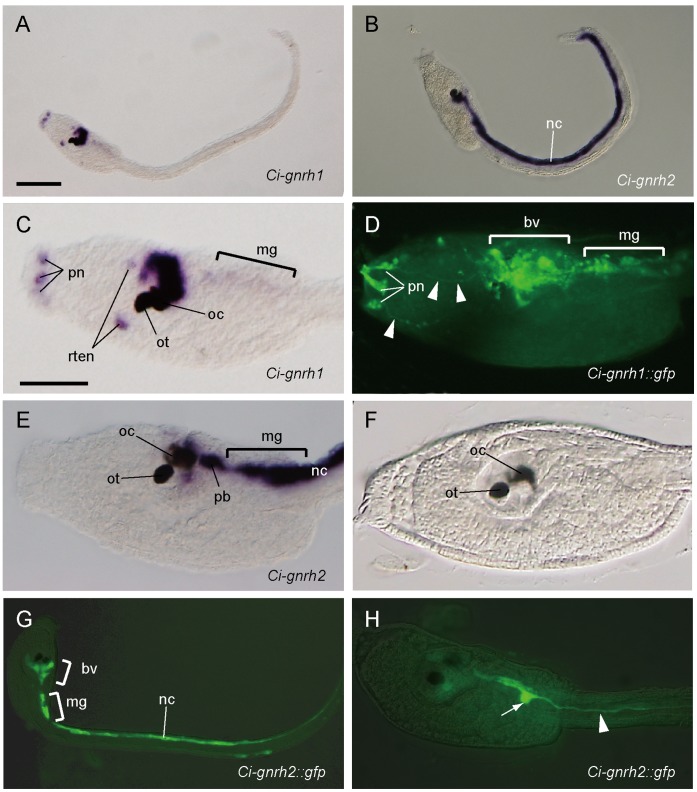
Expression of *gnrh* genes in the *Ciona intestinalis* larvae. Spatial expression patterns of *Ci-gnrh1* (A, C, and D) and *Ci-gnrh2* (B, E, G, and H) in a whole body (A, B, and G) or trunk region (C-F, and H) of *C. intestinalis* larvae. The gene expression was visualized by whole-mount *in situ* hybridization (A, B, C, and E) or by GFP reporter expression under the control of the regulatory region of *Ci-gnrh1* (D, G, and H). (F) An unstained control larva showing the otolith (ot) and ocellus (oc) pigment cells in the brain vesicle. White arrowheads in (D) indicate GFP-positive axons of papillar neurons (pn). The white arrowhead in (H) indicates a GFP positive neurite. **bv**, brain vesicle; **nc**, nerve cord; **rten**, rostoral trunk epidermal neuron; **mg**, motor ganglion. Scale bars: 100 µm in (A); 50 µm in (C).

Six GnRH peptides, tGnRH-3 to -8, are encoded by two genes, *Ci-gnrh1* and *Ci-gnrh2* in *C. intestinalis* (Fig. S1) [19]. In previous studies, localization of GnRHs or their mRNAs in ascidians were examined in adult tissues [17], [19], [24]–[26]. Expression of the *Ci-gnrh1* and *Ci-gnrh2* genes has been demonstrated during larval development by RT-PCR [19], but their spatial expression patterns have not been reported. To elucidate potential sites utilizing GnRH signaling in ascidian larvae, we examined the spatial expression patterns of *Ci-gnrh1* and *Ci-gnrh2* in *C. intestinalis* larvae by whole-mount *in situ* hybridization. Both genes were found to be specifically but distinctly expressed in the nervous system of the larvae (Fig. 1A, B). *Ci-gnrh1* is strongly expressed in a population of cells in the brain vesicle, the anterior portion of the central nervous system (Fig. 1A, C). Transcripts of *Ci-gnrh1* were also detected in the motor ganglion (also called “visceral ganglion”; we adopt here a terminology recently proposed by Nishino et al. [27]), but its expression level was much lower than that in the brain vesicle (Fig. 1C). In addition to the expression in the central nervous system, *Ci-gnrh1* is also expressed in parts of the peripheral nervous system: it is expressed in a few cells in the adhesive organ and a subpopulation of rostral epidermal neurons (RTENs) (Fig. 1C). The cells expressing *Ci-gnrh1* in the adhesive organ are likely to be papillar neurons, as they possess long neurites (probably axons) as visualized by GFP under the control of the *cis*-regulatory region of *Ci-gnrh1* (Fig. 1D). The *Ci-gnrh1::gfp* fusion construct could also visualize the brain vesicle and the motor ganglion (Fig. 1D).

The expression of *Ci-gnrh2* was detected mostly in the central nervous system. The most conspicuous expression site of *Ci-gnrh2* was the caudal nerve cord; it was expressed throughout the entire nerve cord from the posterior trunk to the tail tip (Fig. 1B). The brain vesicle and the motor ganglion also contained cells expressing *Ci-gnrh2* (Fig. 1B, E). In the brain vesicle, there are at least two populations of *Ci-gnrh2-*expressing cells: one is located close to the ocellus pigment cell, while the other is located in the posterior brain region (Fig. 1E). Expression of GFP under the control of the *cis*-regulatory region of *Ci-gnrh2* recapitulated the endogenous gene expression pattern (Fig. 1G). In some specimens, *Ci-gnrh2::gfp* visualized a neuron in the motor ganglion, which had a neurite (presumably an axon) extending into the tail along the nerve cord (Fig. 1H).

### Four GnRH Receptor Paralogs in *C. intestinalis*

**Figure 2:**
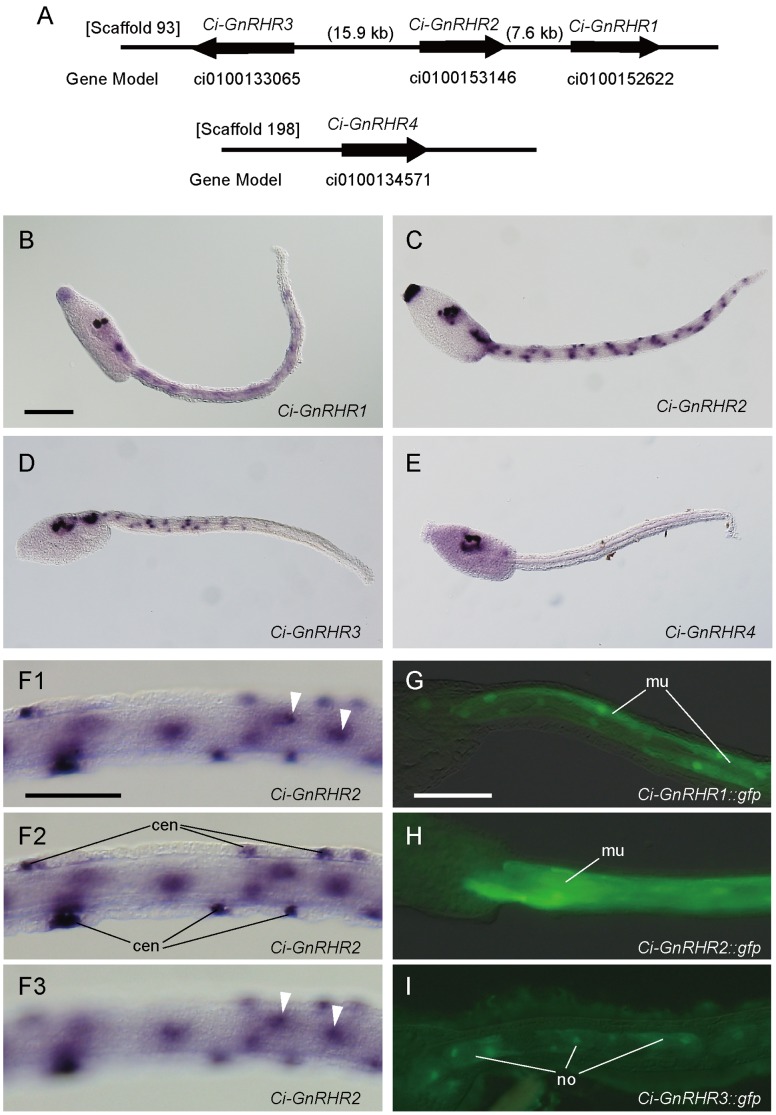
The *C. intestinalis* GnRH receptors (GnRHRs) and spatial expression patterns of *gnrhr* genes in the larva. (A) Genomic organization of the *C. intestinalis* GnRHRs. Three GnRHRs (Ci-GnRHR1, Ci-GnRHR2, and Ci-GnRHR3) are encoded by a gene cluster located on Scaffold 93 while the gene encoding another GnRHR (Ci-GnRHR4) is located in Scaffold 198. Arrows indicate the location and orientation of the genes. Gene model IDs in the JGI version 1.0 genome database are indicated below the arrows. (B-E) Whole larvae showing localization of transcripts of *Ci-GnRHR1* (B), *Ci-GnRHR2* (C), *Ci-GnRHR3* (D), or *Ci-GnRHR4* (E). (F1–F3) A middle part of the tail of the same individual with different focal planes showing muscle cells and caudal epidermal neurons (cen) expressing *Ci-GnRHR2*. (G-I) GFP reporter expression in the tail muscle (mu) or notochord (no) under the control of the regulatory region of *Ci-GnRHR1* (G), *Ci-GnRHR2* (H), or *Ci-GnRHR3* (I). **cen**, caudal epidermal neuron; **mu**, muscle; **no**, notochord. Scale bars: 100 µm in (B), 50 µm in (F1) and (G).

Four GnRH receptors (Ci-GnRHR1, Ci-GnRHR2, Ci-GnRHR3, and Ci-GnRHR4) are encoded in the genome of *C. intestinalis* [20], [21] (Fig. 2A). We previously identified and characterized Ci-GnRHR1 and Ci-GnRHR2 [20], and Tello et al. [21] reported identification of Ci-GnRHR3 and Ci-GnRHR4 along with functional characterization of the four *C. intestinalis* GnRH receptors. In this study, we independently isolated full-length cDNA clones for Ci-GnRHR3 and Ci-GnRHR4. The *Ci-GnRHR4* cDNA clone we isolated contained an additional exon at the 3′ end, resulting in an extended sequence coding for a 411 amino acids polypeptide as compared to the previously reported 366 amino acids polypeptide [21]. The positions of introns, including this newly identified intron, are conserved among all *C. intestinalis* GnRH receptor genes (Fig. S2). Another new finding is that the 5′ ends of the *Ci-GnRHR3* and *Ci-GnRHR4* cDNAs have a spliced leader sequence (5′-ATTCTATTTGAATAAG-3′), which is identical to that found in the *C. intestinalis* troponin I gene [28]. The nucleotide sequences of the *Ci-GnRHR3* and *Ci-GnRHR4* cDNAs have been deposited in the DDBJ, EMBL, and GenBank Nucleotide Databases under the accession numbers AB540990 and AB540991, respectively.

There are a number of amino acid differences between the previously reported sequences of Ci-GnRHR3 and Ci-GnRHR4 obtained from a north Atlantic population in Woods Hole, MA [21] and the present sequences obtained from a Pacific population in Japan; eighteen and 7 amino acids were different in Ci-GnRHR3 and Ci-GnRHR4, respectively (Fig. S2). Similar extent of sequence divergence between North Atlantic and Pacific populations was also reported for Ci-GnRHR1 and Ci-GnRHR2 [21] (Fig. S2). Recent studies have revealed the existence of two cryptic species: *C. intestinalis* sp. A, distributed in the Mediterranean, northeast Atlantic, and Pacific, and *C. intestinalis* sp. B, found in the North Atlantic [29]. Thus, the observed divergence between the two sets of Ci-GnRHR sequences is probably attributable to the genetic variations between *C. intestinalis* sp. A and *C. intestinalis* sp. B.

### Molecular Phylogeny of the GnRHR Family

Roch and colleagues recently reported a large-scale phylogenetic analysis of GnRHRs [30]. We also examined the phylogenetic relationships among GnRHRs, and compared our results with the previous study [30]. The protein dataset analyzed consisted of 55 receptor sequences, including two new lamprey GnRHRs, and the human oxytocin and vasopressin V2 receptors, used as outgroups (Table S1). Multiple sequence alignment was performed using TM-Coffee [31], which is specifically designed to align transmembrane proteins. Only the aligned residues with a high confidence index were retained (see Materials and Methods). Maximum Likelihood (ML) phylogenetic inference was carried out using PhyML v3 [32], Leaphy v1.0 [33] and RAxML v7.2.8 [34], for these programs use different tree-space search strategies. The three strategies converged on the same topology, which is shown in Fig. S3. See Materials and Methods for details.

Our results are largely consistent with the previous report [30]. First, GnRH receptors form a superfamily with corazonin (Crz) and adipokinetic hormone (AKH) receptor families. Second, the four GnRH receptors in *C. intestinalis* belong to a paralogous cluster (possibly tunicate-specific). Third, there are two distinct lineages of amphioxus GnRH receptors [35], one (amphioxus GnRHR1 and 2) closely related to the vertebrate and tunicate GnRH receptors and the other (amphioxus GnRHR3 and 4) grouped together with protostome GnRH and corazonin receptors. Fourth, diversification of GnRH receptors occurred independently in each chordate subphylum, although we could not statistically resolve interrelationships among vertebrate, tunicate, and cephalochordate GnRHRs.

A major difference between the present result and the tree by Roch et al. [30] is the position of sea urchin GnRHRs. These authors proposed that sea urchin GnRHRs are orthologs of CrzRs/GnRHRs. In contrast, in our analysis, the three sea urchin receptors lie basal to the chordate clade. We consider this topology more likely than the alternative topology (i.e., GnRHR + AKHR/ACPR clade, Fig. 5 in [30]) given the high log-likelihood difference (ΔlogL = 9.43) between our topology and the one proposed by Roch et al., though the latter cannot be excluded on statistical grounds (see Materials and Methods).

### *Ciona GnRHRs* are Expressed in Phylotypic Axial Structures

The conspicuous expression of *Ci-gnrh1* and *Ci-gnrh2* in the larvae suggests that their receptors are also present and function at the larval stage. In previous studies, however, spatial expression patterns of genes encoding Ci-GnRHRs was only examined in adult tissues [20], [21]. We, therefore, examined spatial expression patterns in the larva of *Ci-GnRHR1*, *Ci-GnRHR2*, *Ci-GnRHR3*, and *Ci-GnRHR4* by whole-mount *in situ* hybridization. All *Ci-GnRHR* genes are expressed in the brain vesicle (Fig. 2B, C, D, E). *Ci-GnRHR1*, *Ci-GnRHR2*, and *Ci-GnRHR3* are also expressed in the motor ganglion (Fig. 2B, C, D). In addition, *Ci-GnRHR1* and *Ci-GnRHR2* are expressed in the adhesive organ and the tail muscle cells (Fig. 2B, C, F). *Ci-GnRHR2* is also expressed in some cells located on the dorsal and ventral midlines of the tail (Fig. 2C, F). These midline cells seem to be caudal epidermal neurons, which express a glutamatergic marker *Ci-VGLUT* [36]. *Ci-GnRHR3* is also expressed in the tail, but the expression pattern is different from those of *Ci-GnRHR1* and *Ci-GnRHR2*; its expression is more evident in the proximal (anterior) than distal region of the tail (Fig. 2D). The cells expressing *Ci-GnRHR3* in the tail are distributed along the cavity of the notochord in a pattern similar to that of nuclei in the mature notochord [37]. The expression of *Ci-GnRHR1* and *Ci-GnRHR2* in muscle cells and *Ci-GnRHR3* in notochord cells was further supported by *GFP* reporter expression under the control of the *cis*-regulatory region of each gene (Fig. 2G, H, I). In summary, *Ciona GnRHRs* are conspicuously expressed in phylotypic axial structures, including the central nervous system, notochord, and paraxial muscle.

### Signaling and Regulation of *Ciona* GnRHRs

We confirmed the previous report [21] that in COS-7 cells Ci-GnRHR1 activates IP generation in response to tGnRH-6 (Table S2). We also found that tGnRH-7 and tGnRH-8 were able to stimulate IP accumulation in COS-7 cells expressing Ci-GnRHR1, but yet with relatively high EC_50_ values (Table S2). In HEK293-MSR cells expressing Ci-GnRHR1, in which elevation of intracellular Ca^2+^ is induced with much lower concentrations of tGnRHs than IP generation in COS-7 cells [38], tGnRH-6 evoked the most potent elevation of intracellular Ca^2+^, whereas other tGnRHs showed 10-fold to 1000-fold lower activities (Table 1; Fig. S4A). No significant Ca^2+^ increase was observed in cells expressing Ci-GnRHR2, 3, or 4 in response to any tGnRHs. These results confirmed that Ci-GnRHR1 exclusively stimulates mobilization of intracellular Ca^2+^ with high specificity to tGnRH-6.

**Table 1:** EC_50_ values (nM) of tGnRHs for intracellular calcium ion mobilization in HEK293-MSR cells expressing only Ci-GnRHR1 or co-expressing Ci-GnRHR1 and 4.

	Ci-GnRHR1	Ci-GnRHR1&4
tGnRH-3	>100	>100
tGnRH-4	>100	>100
tGnRH-5	>100	>100
tGnRH-6	6.971	0.55
tGnRH-7	100	88.8
tGnRH-8	>100	>100

The EC_50_ values of tGnRH-6 are underlined to emphasize the dramatic increase in the potency on the cells co-transfected with Ci-GnRHR1 and 4.

Activation of the cAMP pathway by tGnRHs has been reported as another cellular response in COS-7 cells expressing Ci-GnRHRs [21]. We examined induction of cAMP production by each combination of tGnRH and Ci-GnRHR in HEK293-MSR cells. Our results obtained using HEK293-MSR cells (Table 2; Fig. S4B, C, D) are compatible with the previous report using COS-7 cells [21], indicating the ligand-selective cAMP production mediated by Ci-GnRHR1, Ci-GnRHR2, and Ci-GnRHR3. We also confirmed that Ci-GnRHR4 was devoid of cAMP production or inhibition upon application of any tGnRHs (data not shown).

**Table 2:** EC_50_ values (nM) of t-GnRHs for cAMP production in HEK293-MSR cells expressing only Ci-GnRHR1, 2, 3 or co-expressing Ci-GnRHR1 and 4, or Ci-GnRHR3 and 4.

	Ci-GnRHR1	Ci-GnRHR2	Ci-GnRHR3	Ci-GnRHR1&4	Ci-GnRHR3&4
tGnRH-3	>100	>100	19.5	>100	19.1
tGnRH-4	>100	>100	>100	>100	>100
tGnRH-5	>100	>100	9.19	>100	6.09
tGnRH-6	0.334	>100	>100	0.318	>100
tGnRH-7	34	29.2	>100	27.6	>100
tGnRH-8	31	8.78	>100	19.9	>100

No markedly altered effects of co-expression with Ci-GnRHR4 on activities of tGnRHs were detected.

We recently demonstrated that Ci-GnRHR4 could form a heterodimer with Ci-GnRHR1 and Ci-GnRHR2 *in vivo* and modulate signaling via these receptors [38], [39]. Here we further evaluated the specificity of the modulator function of Ci-GnRHR4. In the cells co-transfected with Ci-GnRHR1 and Ci-GnRHR4, tGnRH-6 exhibited approximately 10-fold more potent activity on intracellular calcium mobilization than in the cells expressing Ci-GnRHR1 alone, whereas the activities of other tGnRHs were not up-regulated (Fig. S4E and Table 2). In contrast, co-expression of Ci-GnRHR1 and Ci-GnRHR4 resulted in no significant alteration of pharmacological profiles of cAMP production, although the maximal levels of cAMP by tGnRH-6 and tGnRH-8 were slightly increased (Fig. S4F). In cells co-expressing Ci-GnRHR3 and Ci-GnRHR4, cAMP production did not differ in potency, magnitude, or ligand selectivity, compared to cells expressing Ci-GnRHR3 alone (Fig. S4G and Table 2). Thus, the regulatory function of Ci-GnRHR4 is specific to a ligand, partner receptor, and intracellular signaling pathway.

### Putative GnRH-producing Neurons in the Vertebrate Hindbrain and Spinal Cord

Unexpectedly conspicuous expression of *Ci-gnrh2* in the motor ganglion and the nerve cord motivated us to ask whether similar expression of *gnrh* genes are found in vertebrates. To the best of our knowledge, however, the GnRH-producing cell bodies have not been reported in the vertebrate hindbrain and spinal cord. We adopted medaka *O. latipes* as a vertebrate model because both its GnRHs and their receptors have been well characterized [40], [41] and cells expressing genes of interest are easily visualized in living animals by promoter transgenics [42], [43].

**Figure 3:**
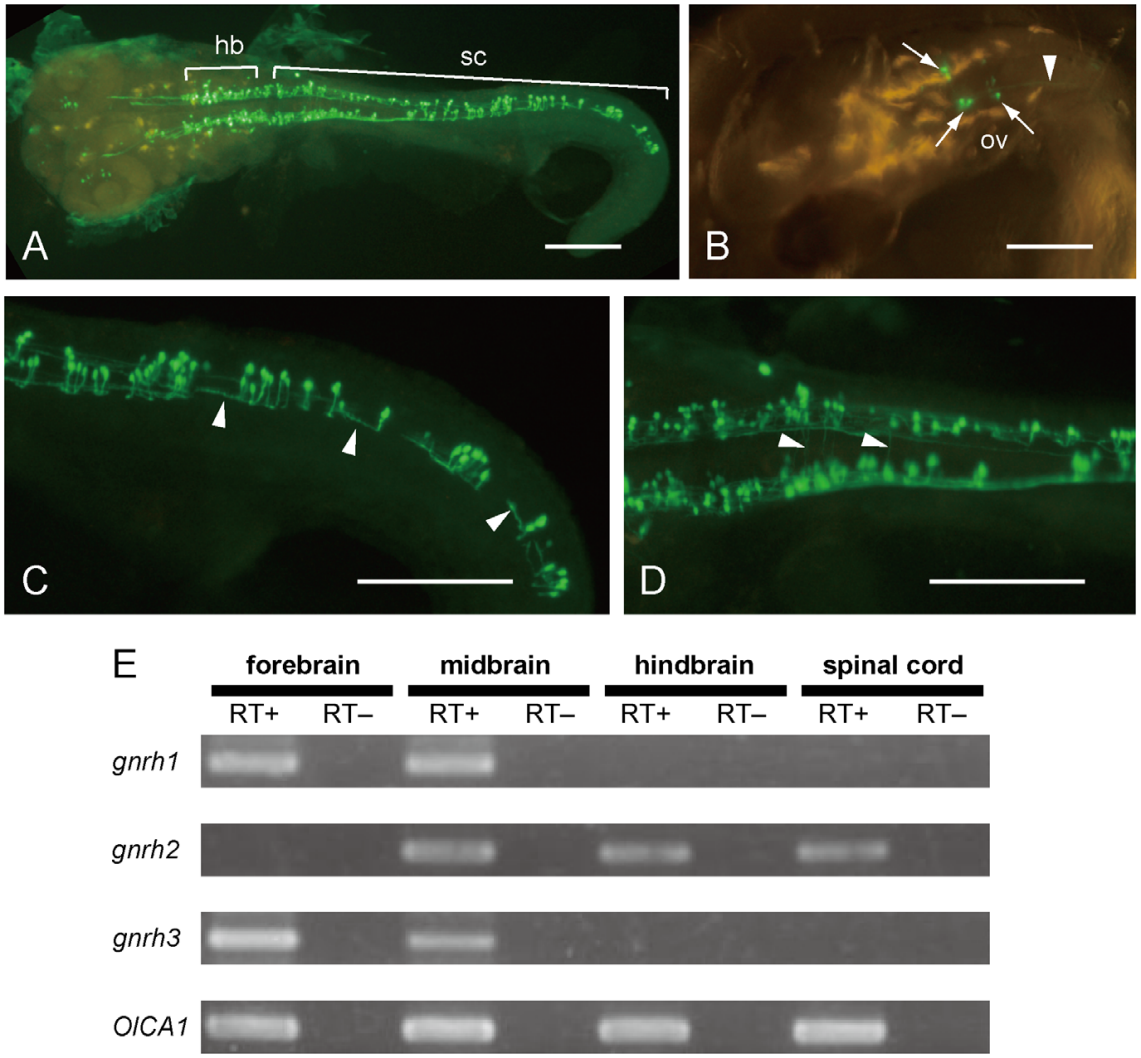
Evidence for expression of a *gnrh* gene in the hindbrain and spinal cord of the medaka *Oryzias latipes*. (A–D) Visualization of cells in the hindbrain and spinal cord with GFP under the control of the *cis*-regulatory region of the medaka *gnrh2* gene. All embryos shown are at 3 days post fertilization (dpf). (A) GFP signals were specifically found in the central nervous system, and were particularly evident in the hindbrain and the spinal cord. (B) An example of embryos showing GFP signal in reticulospinal neurons (arrows) and their axons extending into the spinal cord (arrowheads). (C) Spinal interneurons labeled with GFP showing an axon that first extends ventrally, and turns rostrally in the ventral spinal cord. (D) Some fibers running across the midline were also labeled with GFP, suggesting that some commissural neurons expressed GFP. Scale bars: 200 µm in (A-D). (E) Expression of *gnrh1*, *gnrh2*, and *gnrh3* were detected in different regions of the central nervous system of adult medaka by RT-PCR. The PCR products were analyzed by agarose gel electrophoresis. RT+ and RT– represent amplification with and without reverse transcriptase, respectively. Transcripts of the cytoskeletal actin gene *OlCA1* [42] were amplified as a positive control.

Among the three genes (*gnrh1*, *gnrh2*, and *gnrh3*) encoding GnRH in medaka, we focused on *gnrh2* for the following two reasons. First, embryonic expression has been reported for *gnrh1* and *gnrh3* [44], but not for *gnrh2*. Second, chicken GnRH-II, which is encoded by the medaka *gnrh2* gene [40], can effectively activate Ci-GnRHRs [21]. We isolated a 5-kb upstream region of medaka *gnrh2* and used it to drive the GFP reporter in medaka embryos. Three days after fertilization, GFP fluorescence specifically appeared in the central nervous system, including the hindbrain and spinal cord (Fig. 3A). In the hindbrain, cell bodies of some reticulospinal neurons were labeled with GFP (Fig. 3B). Their axons that extend into spinal cord were also labeled (Fig. 3B). In the spinal cord, at least two types of neurons were labeled. One type includes neurons with a primary axon that first extends ventrally, turns rostrally in the ventral spinal cord, and then ascends on the ipsilateral side of the spinal cord among other axons in the marginal zone (Fig. 3C). The morphology of this type of neurons closely resembles that of V1 inhibitory interneurons [45]–[47]. Labeled neurons of the other type were commissural neurons whose axons extended across the ventral midline towards contralateral sides (Fig. 3D). Neurons of the former type were more abundant among GFP-positive neurons; they distributed throughout the entire spinal cord along the antero-posterior axis. The expression of *gnrh2* in the hindbrain and spinal cord of the medaka was further confirmed by RT-PCR. Transcripts of *gnrh2* were detected in the midbrain, hindbrain and spinal cord, while those of *gnrh1* and *gnrh3* were only detected in the forebrain and midbrain (Fig. 3E). The results suggest that cells expressing a *gnrh* gene are present in the vertebrate hindbrain and spinal cord.

## Discussion

### Complexity of the GnRH System in Tunicates

Two or more GnRH peptides and their receptors have been reported in most animal species studied [48]–[50]. Among these, the tunicate *C. intestinalis* is particularly striking for the multiplicity of GnRH ligands and receptors. In the present study, we showed that the genes encoding all these peptides and receptors were conspicuously expressed in the larvae. Expression patterns of these genes are distinct from each other. The differential expression of paralogous genes adds further complexity to the tunicate GnRH system.

Tello et al. [21] reported selective activation of Ci-GnRHRs by different t-GnRHs, and previous works, we demonstrated that Ci-GnRHR4 could modulate Ci-GnRHR1 and Ci-GnRHR2 signaling via heterodimerization [38], [39]. The present study confirmed these previous findings and further extended the understanding of the selectivity and specificity of these molecular interactions. Expression patterns of *Ci-GnRHR* and *Ci-gnrh* genes are consistent with receptor-ligand selectivity. For example, Ci-GnRHR1 and Ci-GnRHR2 in muscle cells can be activated by tGnRH-7 and tGnRH-8, which are encoded by *Ci-gnrh2* expressed in the nerve cord. *Ci-gnrh1* encodes tGnRH-6, which is the most potent ligand for Ci-GnRHR1 and a moderate activator for Ci-GnRHR2; consistently, *Ci-gnrh1*, *Ci-GnRHR1*, and *Ci-GnRHR2* are all expressed in the adhesive organ and in the brain vesicle. *Ci-gnrh1* also encodes tGnRH-3 and tGnRH-5, potential ligands for Ci-GnRHR3. *Ci-GnRH3* is expressed in the brain vesicle, motor ganglion, and anterior notochord, which are reasonably close to the expression sites of *Ci-gnrh1*. The consistency between expression patterns and effective ligand-receptor combinations suggests the functional significance of GnRH signaling in the ascidian larva.

Ci-GnRHR4 is a positive regulator for Ci-GnRHR1 in the generation of intracellular calcium ions by interaction with tGnRH-6. The action of Ci-GnRHR4 is specific with respect to heterodimer partners, ligands, and intracellular signaling pathways. Superimposition of possible sites of Ci-GnRHR1 modulation by Ci-GnRHR4 onto the expression patterns of the two receptors can increase the regulatory complexity of GnRH signaling in the ascidian larva. For example, *Ci-GnRHR1* and *Ci-GnRHR4* are co-expressed in the brain vesicle, whereas only *Ci-GnRHR1* is expressed in the adhesive organ, motor ganglion, and muscle cells. Thus, ligand-receptor selectivity, differential gene expression, and receptor heterodimerization generate a vast diversity of GnRH signaling modes at different sites of the larva.

### Non-reproductive Features of the GnRH System in *Ciona*

The larvae of *C. intestinalis* occupy a non-reproductive dispersal stage; the gonad develops after metamorphosis and reproduction occurs much later [51], [52]. During metamorphosis, the nervous system undergoes extensive reorganization [53] and the tail, containing the nerve cord, muscle, notochord, and caudal epidermis, degenerates [54], [55]. Conspicuous expression of the genes for GnRHs and GnRHRs in the nervous system and tail tissues in the larva strongly suggests that GnRHs regulate biological processes that are not directly related to reproduction. What kind of biological processes do they regulate? Regulation of locomotion and swimming behavior is one possibility. Two types of environmental signal, light and gravity, regulate the swimming behavior of the larva [56]–[59]. Light and gravity are sensed by the ocellus and otolith, respectively, in the brain vesicle, and the sensory inputs are then processed in the posterior brain vesicle and motor ganglion to regulate muscle contraction in the tail [27], [60]. Since the receptor genes are expressed in the brain vesicle, motor ganglion, and muscle cells, and the ligand genes are expressed in the brain vesicle, motor ganglion and nerve cord, GnRH signaling would be able to regulate sensory reception, neuronal processing, and muscle functions.

Another possible role of GnRH in the ascidian larva may be the control of metamorphosis. The initiation of metamorphosis is triggered by settlement on substrates with papillae of the adhesive organ [54], [55], [61]. Stimuli of settlement sensed by the adhesive organ are thought to be transmitted to the central nervous system to proceed with metamorphic events in the trunk and tail tissues. When adhesive papillae of *Ciona* larvae have been removed, tail absorption, a major metamorphic event, is suppressed and the tail retains its swimming ability [55]. *Ci-gnrh1* is expressed in papillar neurons, which are potential sensors for metamorphic signals, while *Ci-gnrh2* is expressed in the central nervous system, including the brain vesicle, the visceral ganglion, and the nerve cord, which potentially convey and deliver metamorphic signals to the tail. Consistently, the genes for GnRH receptors are expressed in potential targets of metamorphic signaling, including the adhesive organ, brain vesicle, muscle, notochord, and tail epidermis.

### Evolutionary Conservation of a Non-reproductive GnRH System in Chordates

We found that *cis*-regulatory DNA of the medaka *gnrh2* gene specifically drives transcription in neurons in the hindbrain and spinal cord of medaka embryos. The expression pattern of *gnrh-2::gfp* in medaka embryos is reminiscent of the expression pattern of the *gnrh* genes in the ascidian larva. We further confirmed the expression of endogenous *gnrh2* in the hindbrain and spinal cord by RT-PCR. In the hindbrain, *gnrh-2::gfp* was expressed in reticulospinal neurons, which control spinal locomotor circuits [64]–[66]. In the spinal cord, *gnrh-2::gfp* was expressed in at least two distinct types of neurons: commissural interneurons and a group of ipsilaterally projecting interneurons. Commissural interneurons provide reciprocal coordination between the left and right sides of the spinal cord as parts of central pattern generators (CPGs), the neural networks generating rhythmic movements such as swimming and walking [65]–[68]. The ipsilaterally projecting interneurons expressing *gnrh-2::gfp* have been found to be morphologically similar to the V1 spinal neurons. The V1 interneurons are inhibitory neurons that innervate motor neurons and play an important role in the locomotor CPG [45]–[47]. Thus, GnRH signaling may regulate the function or development of neural circuits that generate rhythmic movements in vertebrates.

In *Ciona* larvae, the locomotor CPG has been proposed in the motor ganglion and the anterior nerve cord [27], [60], where the *gnrh* genes are also expressed. Homology between the tunicate motor ganglion/nerve cord and the vertebrate hindbrain/spinal cord has been suggested by developmental studies, such as those examining the expressions and functions of conserved transcription factors and signaling molecules [69]–[72]. Taken together with these previous findings, the current data suggest the deep evolutionary origin of a non-reproductive embryonic/larval GnRH system in chordates.

## Materials and Methods

### Ethics Statement

All the treatments of animals in this research followed the Japanese Act on Welfare and Management of Animals (Act No. 105 of October 1, 1973; the latest revisions Act No.68 of 2005, Effective June 1, 2006) and were approved by the Institutional Animal Care and Use Committee of Konan University (approval nos. K-11-04 and K-10-02). No specific permits were required for the described field studies.

### Animals and Embryos

Mature adults of *C. intestinalis* were collected from harbors in Murotsu and Aioi, Hyogo, Japan, or obtained from the National BioResource Project (NBRP) and maintained in indoor tanks of artificial seawater (Marine Art BR, Senju Seiyaku, Osaka, Japan) at 18°C. Embryos and larvae were prepared as described previously [73].

Mature adults of the orange-red variety of medaka *Oryzias latipes* were a gift from Dr. Rie Kusakabe (Kobe University). They were kept in indoor tanks under artificial reproductive conditions (9 h 45 min dark, 14 h 15 min light; 28°C) and fed on Otohime B2 artificial feed (Marubeni Nisshin Feed Co., Ltd., Tokyo, Japan). Naturally spawned and fertilized eggs were collected and the embryos were cultured in distilled water containing 0.6-ppm methylene blue at 28°C. Embryos were dechorionated using hatching enzyme [74] provided by the NBRP-Medaka.

### Isolation and Characterization of cDNAs Encoding *C. intestinalis* GnRHRs

The genes encoding Ci-GnRHR3 and Ci-GnRHR4 were found in the *C. intestinalis* genome database [75] by TBLASTN searches using amino acid sequences of human and *C. intestinalis* GnRHRs as queries. A 1231- or 751-bp cDNA fragment encoding a portion of Ci-GnRHR3 or Ci-GnRHR4 was amplified from a mid tailbud cDNA pool by PCR with a pair of primers (5′-CAACACTATTCAACTAACGCGAG-3′ and 5′-AGTATAGTTGAACCAGGATTATTACGTC-3′ for *Ci-GnRHR3*; 5′-CGTATTTGGGTCACGTGG-3′ and 5′-CCAACAAATAACGAATGTGATGG-3′ for *Ci-GnRHR4*). The 5′ end of *Ci-GnRHR3* mRNA was determined by the 5′ RACE method using a Gene Racer kit (Invitrogen). The nucleotide sequences of the primers used for 5′RACE were 5′-CCATGTTGCGTCCATTGGCATGCT-3′ (for the primary PCR) and 5′-GGTGATGACGTGGACATGAAGTCTCT-3′ (for the nested PCR). The full-length coding sequence of *Ci-GnRHR3* cDNA was amplified by PCR from a cDNA pool of mid tailbud embryos using gene-specific primers (forward primer 5′-GAAACCTGTCATAAGACTTGTTAGT-3′; primary reverse primer 5′-AGTATAGTTGAACCAGGATTATTACGTC-3′; nested reverse primer 5′-ATGGTGACGCCACGATG-3′). The 5′ end and 3′ end of *Ci-GnRHR4* mRNA were determined by the 5′ RACE and 3′ RACE methods, respectively, using a Gene Racer kit (Invitrogen). The primer sequences used for 5′ RACE were 5′-GCGTAATAACCCGGTGACCCTGTCTAC-3′ (for the primary PCR) and 5′-ACCTGCTAACCACTCAAGCGTGTA-3′ (for the nested PCR). The primer sequences used for 3′ RACE were 5′-GTCCCAACCATTATGTGGTGCAA-3′ (for the primary PCR) and 5′-GAAGAGGGCAAAAGCAACCGAACT-3′ (for the nested PCR). The full-length coding sequence of *Ci-GnRHR4* cDNA was amplified by PCR from a cDNA pool of mid tailbud embryos using gene-specific primers (forward primer 5′-AGGTCTCACAACTAGGTTTGTGG-3′; primary reverse primer 5′-GACGACACAGATCACACATGAC-3′; nested reverse primer 5′-CTGAGAAGCGCACAGACTC-3′). The cDNA fragments were cloned into a pBluescript II SK(+) vector. The cDNA clones were sequenced on both strands by the cycle sequencing method with an Applied Biosystems 3100 Genetic Analyzer (Applied Biosystems, Foster City, CA, USA).

### Molecular Phylogenetics

The protein dataset analyzed consisted of 55 receptor sequences as well as the human oxytocin and vasopressin V2 receptors, used as outgroups (Table S1). Sequences were retrieved from Uniprot, GenBank/NCBI and Joint Genome Institute (JGI) web sites. Multiple sequence alignment was performed using TM-Coffee [31]. The per-site confidence of aligned residues was assessed using the CORE index (part of the T-Coffee suite [76]). The CORE index reflects the consistency of aligned residues with respect to the TM-Coffee alignment library; only residues with CORE index 8–9 (on a 0–9 scale) were retained. The alignment was inspected manually and edited using SeaView [77]. The final alignment (available upon request) consisted of 243 sites. ProtTest v3.2 [78] identified JTT +Γ4+F as the best model of protein evolution that fit the data, according to all the statistics implemented; this model was used to infer the ML trees. In order to better explore the tree-topology space, we used PhyML v3 (build 20120412) [32], Leaphy v1.0 [33] and RAxML v7.2.8 [34]. Tree-topology searches in PhyML were conducted using the Subtree Pruning and Regrafting moves (starting from 10 random trees and the BioNJ tree). Branch support values were estimated in PhyML by 100 non-parametric bootstrap replicates and by the less conservative parametric aBayes statistic (Bayesian-like transformation of approximate likelihood ratio test [79]).

As far as the position of the sea urchin receptors is concerned, we reconstructed manually the topology proposed by Roch et al. in [30], and compared it with the one obtained in the present analysis. Their statistical confidence was assessed using the SH [80], KH [81] and ELW [82] tests as implemented in TreePuzzle v5.2 [83].

### Whole-mount *in situ* Hybridization

For synthesizing the *Ci-GnRHR1* and *Ci-GnRHR2* probes, the full-length cDNA clones previously reported [20] were used. For *Ci-GnRHR3* and *Ci-GnRHR4* probes, cDNA clones containing the full-length coding region for *Ci-GnRHR3* and *Ci-GnRHR4* described above were used. A *Ci-gnrh1* cDNA fragment was amplified from a larval cDNA pool by PCR with a pair of gene-specific primers (5′-GCAATCGCTTATCCAACTTCAC-3′ and 5′-GCATAAAGCGTGCACACAAG-3′), and subcloned into pBluescriptII SK(+). The cDNA clone for *Ci-gnrh2* (Gene Collection ID GC27a12) was obtained from the *Ciona* Gene Collection release 1 [84]. The plasmid clones were digested with a restriction enzyme, and used as the template to synthesize a digoxigenin-labeled antisense RNA probe using a DIG RNA labeling kit (Roche, Japan). *Ciona intestinalis* embryos and larvae were fixed in 4% paraformaldehyde in 0.1 M MOPS (pH 7.5) and 0.5 M NaCl at 4°C for 16 hr, prior to storage in 80% ethanol at -30°C. Whole-mount *in situ* hybridization was carried out as described [85].

### Labeling Cells with Green Fluorescent Protein (GFP) Using *cis*-regulatory Regions of *gnrh* or *GnRHR* Genes

The 5′ flanking sequences of *Ci-gnrh1*, *Ci-gnrh2*, *Ci-GnRHR1*, *Ci-GnRHR2*, and *Ci-GnRHR3* obtained from the JGI Genome Database were used to design gene-specific primers. Genomic DNA fragments containing an upstream region of each gene were amplified from sperm DNA by PCR using a thermostable DNA polymerase (LA Taq, Takara BIO, Japan) and a pair of gene-specific oligonucleotide primers (5′-CAAGAGCTCTGCTGTAACACACGCATGTT-3′ and 5′-CAACCCGGGATTGTGAAGTTGGATAAGCGA-3 for *Ci-gnrh1*; 5′-TGACGTCGACAGGAGCAGACGTCATAAGTA-3′ and 5′-ACGGATCCTGTTACGTTATCTCTCTAGAAG-3 for *Ci-gnrh2*; 5′-CAAGAGCTCGAATATGTGGTAGTCAGCAA-3′ and 5′-TGACGTCGACATGCACTGTTCGCTTATGAT-3 for *Ci-GnRHR1*; 5′-CAAGAGCTCGGCTGAACGGGACATCATTA-3′ and 5′-CAACCCGGGCTATACGACTCAGATCGTTC-3 for *Ci-GnRHR2*; and 5′-CAAGAGCTCGGTTAAGCAAACTGTTAGCG-3′ and 5′-CAACCCGGGAGGTTTGACTCACTAACAAG-3 for *Ci-GnRHR3*). The amplified upstream regions were inserted into the *Sac*I/*Sma*I (*Ci-gnrh1*, *Ci-GnRHR2*, and *Ci-GnRHR3*), *Sal*I/*Bam*HI (*Ci-gnrh2*), or *Sac*I/*Sal*I (*Ci-GnRHR1*) sites of pBluescript-EGFP [86]. The sizes of the genomic DNA fragments inserted were 4.7 kb (*Ci-gnrh1*), 4.3 kb (*Ci-gnrh2*), 5.1 kb (*Ci-GnRHR1*), 4.6 kb (*Ci-GnRHR2*), and 5.7 kb (*Ci-GnRHR3*). The promoter-*GFP* fusion construct was electroporated into fertilized eggs of *C. intestinalis* as described by Corbo et al. [87]. The reporter expression was observed as described previously [36].

The 5′ flanking sequence of the medaka *gnrh2* gene obtained from the Ensembl genome database was used to design gene-specific primers. The *O. latipes* genomic DNA was extracted from one individual of the Hd-rR inbred strain as described previously [43]. Genomic DNA fragments containing an upstream region of *gnrh2* were amplified from the genomic DNA by PCR using a thermostable DNA polymerase (PrimeStar HS DNA polymerase, Takara BIO, Japan) and a pair of gene-specific oligonucleotide primers, 5′-GACTGTCGACATCAGGCTTGCTTTGTTG-3′ and 5′-GACTGGATCCCGAGACATTACCTAAAACC-3. The amplified 5-kb upstream region was inserted into the *Sal*I/*Bam*HI sites of pBluescript-EGFP. The resultant plasmid DNA was introduced into medaka embryos by microinjection, and GFP fluorescence was observed as described previously [42].

### RT-PCR Analysis

Total RNA was isolated from the forebrain, midbrain, hindbrain, and spinal cord of an adult medaka using ISOGEN (Nippon Gene Co., Ltd., Tokyo, Japan). One µg of total RNA was used as the template to synthesize the first strand cDNA using an oligo(dT) primer according to the manufacturer’s protocol (SuperScript™ III First Strand Synthesis System for RT-PCR; Invitrogen Corp., Carlsbad, CA). The cDNA fragment of *gnrh1*, *gnrh2*, *gnrh3* or cytoskeletal actin *OlCA1* [42], which was used as a control, was amplified by PCR from the first-strand cDNA. The primer sets used were as follows: for *gnrh1*, 5′-ACTTATGAACTGAAGCTCTGTGT-3′ and 5′-TTACACTCCCAAGAAGCCC-3′; for *gnrh2*, 5′-ACCACTCAGACTAAGGTAATGTC-3′ and 5′-CAAGGCATCCAGAACAATATTTCT-3′; for *gnrh3*, 5′-GAAAACACAGAGTTCTAATGGACG-3′ and 5′-GAGCCATCATTAATTAGATTGTATTGTC-3′; and for *OlCA1*, 5′-GACCCAGATCATGTTTGAGACT-3′ and 5′-GACTCCATACCAAGGAAGGAA-3′. After denaturation at 98°C for 2 min, the PCR was performed for 35 cycles [98°C for 10 sec, 55°C for 30 sec, and 72°C for 1 min] followed by a final extension at 72°C for 7 min using 5% (1.0 µl) of the cDNA reaction mixture as a template, 25 pmol of each primer, and 1.25 U of a Taq DNA polymerase (Ex Taq; Takara Bio, Japan). To eliminate the possibility of PCR amplification derived from contaminated genomic DNA, 35 cycles of amplification were carried out without reverse transcriptase as a control experiment. The PCR products were separated on a 1.5% agarose gel and stained with ethidium bromide.

### Cell Culture, Plasmid Construction, and Transfection

The full-length open reading frame of each *Ci-GnRHR* cDNA was cloned into a pcDNA3.1 plasmid vector. COS-7 cells and HEK293-MSR cells were purchased from American Type Culture Collection (ATCC; Rockville, MD, U.S.A.) and Invitrogen Japan (Tokyo, Japan), respectively. COS-7 cells or HEK293-MSR cells were grown under 5% CO_2_ and 100% relative humidity in DMEM medium supplemented with 10% (v/v) fetal bovine serum and 0.1 mM non-essential amino acids. Each Ci-GnRHR open reading frame-containing pcDNA 3.1 plasmid or empty vector was transfected into COS-7 cells using Lipofectamine 2000 (Invitrogen, San Diego, CA, U.S.A) or HEK293-MSR cells using Targefect F-1 reagent (Targeting Systems, Santee, CA, U.S.A.) according to the manufacturer’s instructions, and the cells were incubated for 24 hours (inositol phosphate assay) or 48 hours (measurement of intracellular Ca^2+^ and cAMP).

### Inositol Phosphate Assay

Twenty-four hours after transfection, the COS-7 cells were washed twice with Medium 199 (Nacalai Tesque, Kyoto, Japan) and incubated in Medium 199 containing 2% (vol/vol) dialyzed FCS and labeled with 1 µCi [^3^H]myo-inositol with PT6-271 stabilizer (TRK-912, Amersham) per well at 37°C for 48 hours. Medium was then removed, and cells were washed twice with buffer A (140 mM NaCl, 20 mM Hepes, 4 mM KCl, 8 mM D-glucose, 1 mM MgCl_2_, 1 mM CaCl_2_). Then cells were preincubated with buffer A containing 10 mM LiCl for 15 min, followed by addition of the GnRHs at various concentrations at 37°C for 45 min. The reaction was stopped by removing the incubation medium and adding 1 ml of ice-cold 0.6 M perchloric acid and 0.2 mg/ml phytic acid. The samples were then transferred to microcentrifuge tubes, kept on ice for 15 min, and then neutralized by adding 350 µl of 1 M K_2_CO_3_/5 mM EDTA. After neutralization, the samples were centrifuged at 10,000 × g at 4°C for 2 min, and the supernatants were transferred to columns containing AG 1-X8 anion-exchange resin (Bio-Rad). Total inositol phosphates were eluted with 1.05 M ammonium formate/0.1 M formic acid, and radioactivity was determined.

### Determination of Intracellular Calcium Ion and cAMP Levels

For measurement of ligand-induced intracellular calcium ion, HEK293-MSR cells were loaded for 60 min with Ca-3 fluorescent calcium indicator mixture (Molecular Devices, Sunnyvale, CA, U.S.A) 48 hours after transfection. Each t-GnRH ligand at various concentrations was automatically administrated to the cells in a FlexStation II apparatus (Molecular Devices). Real-time fluorescent kinetics at an excitation wavelength of 485 nm and emission wavelength of 525 nm was observed for 3 minutes using a FlexStation II apparatus. Cyclic AMP production was evaluated by a fluorescent competitive immunoassay for cAMP using CatchPointTM Cyclic AMP assay kit (Molecular Devices) and FlexStation II according to the manufacturer’s instructions.

## Supporting Information

Figure S1 **GnRH peptides and** ***gnrh*** **genes in** ***Ciona intestinalis***. (A) Structure of the two *C. intestinalis* genes encoding GnRHs. 5′ is to the left. White boxes indicate untranslated regions (noncoding exon sequences), black boxes indicate coding sequences, and each of the gray boxes with a number (3, 4, 5, 6, 7 or 8) indicates the region encoding a single GnRH peptide shown in (B). Horizontal lines indicate introns and intergenic sequences. The gene model ID for each gene is indicated in parentheses. (B) Primary structure of six *C. intestinalis* GnRHs. “pGlu” refers to the N-terminal pyroglutamic acid, and “Gly-NH2” indicates the C-terminal glycine amide. This figure is modified from [19].
(TIF)

Figure S2 **Amino acid comparison among Ci-GnRHRs.** The deduced amino acid sequences of Ci-GnRHR1, Ci-GnRHR2, Ci-GnRHR3, and Ci-GnRHR4 from a Pacific population (Pac; this study) were aligned with those from an Atlantic population (Atl) [21] using the ClustalW program [88], and the alignment was optimized manually. Dashes indicate gaps introduced in the sequence to optimize the alignment. Amino acid residues showing polymorphism between Pacific and Atlantic sequences are indicated in red. The putative transmembrane domains (TM1-TM7) are indicated by horizontal lines. Triangles indicate positions of introns in the gene encoding each Ci-GnRHR.
(TIF)

Figure S3 **Molecular phylogeny of the GnRHR family.** Maximum-likelihood (ML) trees were computed by using PhyML v3 (build 20120412), Leaphy v1.0 and RAxML v7.2.8, from a dataset comprising 57 taxa (243 sites). Human oxytocin (OXYR) and vasopressin V2 receptors (V2R) are used as outgroups. The evolutionary model was JTT+Γ4+F. Branch support values are ML non-parametric bootstrap replicates/parametric aBayes. Only bootstrap values ≥75%, and aBayes values ≥0.95 are shown. Scale bar indicates the estimated number of substitutions per site. Tree topology is from Leaphy. *It was not possible to decide which one of GnRHR3 or GnRHR4 is more closely related to the GnRHR1/GnRHR2 clade (no statistical support for either topology).
(TIF)

Figure S4 **Signaling activities of various tGnRHs at Ci-GnRHRs expressed in HEK293-MSR cells.** (A) Elevation of intracellular calcium ion mediated by Ci-GnRHR1 in response to tGnRHs. (B) cAMP production mediated by Ci-GnRHR1. (C) cAMP production mediated by Ci-GnRHR-2. (D) cAMP production mediated by Ci-GnRHR3. (E) Elevation of intracellular calcium ion in the cells co-expressing Ci-GnRHR1 and Ci-GnRHR4 in response to tGnRHs. (F) cAMP production in the cells co-expressing Ci-GnRHR1 and Ci-GnRHR4. (G) cAMP production in the cells co-expressing Ci-GnRHR-1 and Ci-GnRHR3. Data represent means ± S.E.M. of three independent experiments.
(TIF)

Table S1 **List of proteins with their accession numbers used for the phylogenetic analysis.**
(XLS)

Table S2 **EC_50_ values (nM) of tGnRHs for inositol phosphate accumulation in COS-7 cells expressing Ci-GnRHR1.**
(DOC)

## References

[1] Yamamoto N (2003) Three gonadotropin-releasing hormone neuronal groups with special reference to teleosts. Anat Sci Int 78: 139–155. 14527128 10.1046/j.0022-7722.2003.00051.x

[2] Okubo K, Nagahama Y (2008) Structural and functional evolution of gonadotropin-releasing hormone in vertebrates. Acta Physiol 193: 3–15. 10.1111/j.1748-1716.2008.01832.x 18284378

[3] Oka Y (2009) Three types of gonadotrophin-releasing hormone neurones and steroid-sensitive sexually dimorphic kisspeptin neurones in teleosts. J Neuroendocrinol 21: 334–338. 19210296 10.1111/j.1365-2826.2009.01850.x

[4] Kanda S, Nishikawa K, Karigo T, Okubo K, Isomae S, et al (2010) Regular pacemaker activity characterizes gonadotropin-releasing hormone 2 neurons recorded from green fluorescent protein-transgenic medaka. Endocrinology 151: 695–701. 20032054 10.1210/en.2009-0842

[5] Dolan S, Evans NP, Richter TA, Nolan AM (2003) Expression of gonadotropin-releasing hormone and gonadotropin-releasing hormone receptor in sheep spinal cord. Neurosci Lett 346: 120–122. 12850563 10.1016/s0304-3940(03)00594-9

[6] Chen CC, Fernald RD (2006) Distribution of two gonadotropin-releasing hormone receptor types in a cichlid fish suggest functional specialization. J Comp Neurol 495: 314–323. 16440293 10.1002/cne.20877

[7] Flanagan CA, Chen CC, Coetsee M, Mamputha S, Whitlock KE, et al (2007) Expression, structure, function, and evolution of gonadotropin-releasing hormone (GnRH) receptors GnRH-R1SHS and GnRH-R2PEY in the teleost, *Astatotilapia burtoni* . Endocrinology 148: 5060–5071. 17595228 10.1210/en.2006-1400

[8] Albertson AJ, Talbott H, Wang Q, Jensen D, Skinner DC (2008) The gonadotropin-releasing hormone type I receptor is expressed in the mouse cerebellum. Cerebellum 7: 379–384. 18592335 10.1007/s12311-008-0038-8

[9] Yamamoto N, Oka Y, Amano M, Aida K, Hasegawa Y, et al (1995) Multiple gonadotropin-releasing hormone (GnRH)-immunoreactive systems in the brain of the dwarf gourami, *Colisa lalia*: immunohistochemistry and radioimmunoassay. J Comp Neurol 355: 354–368. 7636018 10.1002/cne.903550303

[10] Sherwood NM, Wu S (2005) Developmental role of GnRH and PACAP in a zebrafish model. Gen Comp Endocr 42: 74–80. 10.1016/j.ygcen.2005.02.007 15862551

[11] Wu S, Page L, Sherwood NM (2006) A role for GnRH in early brain regionalization and eye development in zebrafish. Mol Cell Endocrinol 257–258: 47–64. 10.1016/j.mce.2006.06.010 16934393

[12] Abraham E, Palevitch O, Ijiri S, Du SJ, Gothilf Y, et al (2008) Early development of forebrain gonadotrophin-releasing hormone (GnRH) neurons and the role of GnRH as an autocrine migration factor. J Neuroendocrinol 20: 394–405. 18208553 10.1111/j.1365-2826.2008.01654.x

[13] Kanaho YI, Enomoto M, Endo D, Maehiro S, Park MK, et al (2009) Neurotrophic effect of gonadotropin-releasing hormone on neurite extension and neuronal migration of embryonic gonadotropin-releasing hormone neurons in chick olfactory nerve bundle culture. J Neurosci Res 87: 2237–2244. 19301422 10.1002/jnr.22051

[14] Ramakrishnan S, Lee W, Navarre S, Kozlowski DJ, Wayne NL (2010) Acquisition of spontaneous electrical activity during embryonic development of gonadotropin-releasing hormone-3 neurons located in the terminal nerve of transgenic zebrafish (*Danio rerio*). Gen Comp Endocrinol 168: 401–407. 20515692 10.1016/j.ygcen.2010.05.009 PMC2922451

[15] Delsuc F, Brinkmann H, Chourrout D, Philippe H (2006) Tunicates and not cephalochordates are the closest living relative of vertebrates. Nature 439: 965–968. 16495997 10.1038/nature04336

[16] Putnam NH, Butts T, Ferrier DE, Furlong RF, Hellsten U, et al (2008) The amphioxus genome and the evolution of the chordate karyotype. Nature 453: 1064–1071. 18563158 10.1038/nature06967

[17] Ohkuma M, Katagiri Y, Nakagawa M, Tsuda M (2000) Possible involvement of light regulated gonadotropin-releasing hormone neurons in biological clock for reproduction in the cerebral ganglion of the ascidian, *Halocynthia roretzi* . Neurosci Lett 293: 5–8. 11065124 10.1016/s0304-3940(00)01481-6

[18] Terakado K (2001) Induction of gamete release by gonadotropin-releasing hormone in a protochordate, *Ciona intestinalis* . Gen Comp Endocrinol 124: 277–284. 11742510 10.1006/gcen.2001.7728

[19] Adams BA, Tello JA, Erchegyi J, Warby C, Hong DJ, et al (2003) Six novel gonadotropin-releasing hormones are encoded as triplets on each of two genes in the protochordate, *Ciona intestinalis* . Endocrinology 144: 1907–1919. 12697698 10.1210/en.2002-0216

[20] Kusakabe T, Mishima S, Shimada I, Kitajima Y, Tsuda M (2003) Structure, expression, and cluster organization of genes encoding gonadotropin-releasing hormone receptors found in the neural complex of the ascidian *Ciona intestinalis* . Gene 322: 77–84. 14644499 10.1016/j.gene.2003.08.013

[21] Tello JA, Rivier JE, Sherwood NM (2005) Tunicate gonadotropin-releasing hormone (GnRH) peptides selectively activate *Ciona intestinalis* GnRH receptors and the green monkey type II GnRH receptor. Endocrinology 146: 4061–4067. 15961566 10.1210/en.2004-1558

[22] Makcie GO, Wyeth RC (2000) Conduction and coordination in deganglionated ascidians. Can J Zool 78: 1626–1639.

[23] Mackie GO, Singla CL (2004) Cupular organs in two species of *Corella* (Tunicata: Ascidiacea). Invertebr Biol 123: 269–281.

[24] Powell JFF, Reska-Skinner SM, Prakash MO, Fischer WH, Park M, et al (1996) Two new forms of gonadotropin-releasing hormone in a protochordate and the evolutionary implications. Proc Natl Acad Sci U S A 93: 10461–10464. 8816823 10.1073/pnas.93.19.10461 PMC38407

[25] Tsutsui H, Yamamoto N, Ito H, Oka Y (1998) GnRH-immunoreactive neuronal system in the presumptive ancestral chordate, *Ciona intestinalis* (Ascidian). Gen Comp Endocrinol 112: 426–432. 9843648 10.1006/gcen.1998.7160

[26] Kavanaugh SI, Root AR, Sower SA (2005) Distribution of gonadotropin-releasing hormone (GnRH) by in situ hybridization in the tunicate *Ciona intestinalis* . Gen Comp Endocrinol 141: 76–83. 15707605 10.1016/j.ygcen.2004.11.012

[27] Nishino A, Okamura Y, Piscopo S, Brown ER (2010) A glycine receptor is involved in the organization of swimming movements in an invertebrate chordate. BMC Neurosci 11: 6. 20085645 10.1186/1471-2202-11-6 PMC2822779

[28] Vandenberghe AE, Meedel TH, Hastings KEM (2001) mRNA 5'-leader trans-splicing in the chordates. Genes Dev 15: 294–303. 11159910 10.1101/gad.865401 PMC312621

[29] Caputi L, Andreakis N, Mastrototaro F, Cirino P, Vassillo M, et al (2007) Cryptic speciation in a model invertebrate chordate. Proc Natl Acad Sci U S A 104: 9364–9369. 17517633 10.1073/pnas.0610158104 PMC1890500

[30] Roch GJ, Busby ER, Sherwood NM (2011) Amphioxus: beginning of vertebrate and end of invertebrate type GnRH receptor lineage. Gen Comp Endocrinol 171: 1–16. 21185290 10.1016/j.ygcen.2010.12.014

[31] Chang J-M, Di Tommaso P, Taly J-F, Notredame C (2012) Accurate multiple sequence alignment of transmembrane proteins with PSI-Coffee. BMC Bioinformatics 13 (Suppl 4)S1. 10.1186/1471-2105-13-S4-S1 PMC3303701 22536955

[32] Guindon S, Gascuel O (2003) PhyML: A simple, fast and accurate algorithm to estimate large phylogenies by maximum likelihood. Syst Biol 52: 696–704. 14530136 10.1080/10635150390235520

[33] Whelan S (2007) New approaches to phylogenetic tree search and their application to large numbers of protein alignments. Syst Biol 56: 727–740. 17849327 10.1080/10635150701611134

[34] Stamatakis A (2006) RAxML-VI-HPC: Maximum Likelihood-based phylogenetic analyses with thousands of taxa and mixed models. Bioinformatics 22: 2688–2690. 16928733 10.1093/bioinformatics/btl446

[35] Tello JA, Sherwood NM (2009) Amphioxus: beginning of vertebrate and end of invertebrate type GnRH receptor lineage. Endocrinology 150: 2847–2856. 19264870 10.1210/en.2009-0028

[36] Horie T, Kusakabe T, Tsuda M (2008) Glutamatergic networks in the *Ciona intestinalis* larva. J Comp Neurol 508: 249–263. 18314906 10.1002/cne.21678

[37] Dong B, Horie T, Denker E, Kusakabe T, Tsuda M, et al (2009) Tube formation by complex cellular processes in *Ciona intestinalis* notochord. Dev Biol 330: 237–249. 19324030 10.1016/j.ydbio.2009.03.015 PMC2841060

[38] Sakai T, Aoyama M, Kusakabe T, Tsuda M, Satake H (2010) Functional diversity of signaling pathways through G protein-coupled receptor heterodimerization with a species-specific orphan receptor subtype. Mol Biol Evol 27: 1097–1106. 20026483 10.1093/molbev/msp319

[39] Sakai T, Aoyama M, Kawada T, Kusakabe T, Tsuda M, et al (2012) Evidence for differential regulation of GnRH signaling via heterodimerization among GnRH receptor paralogs in the protochordate, *Ciona intestinalis* . Endocrinology 153: 1841–1849. 22294747 10.1210/en.2011-1668

[40] Okubo K, Amano M, Yoshimura Y, Suetake H, Aida K (2000) A novel form of gonadotropi-releasing hormone in the medaka, *Oryzias latipes* . Biochem Biophys Res Commun 276: 298–303. 11006121 10.1006/bbrc.2000.3476

[41] Okubo K, Nagata S, Ko R, Kataoka H, Yoshiura Y, et al (2001) Identification and characterization of two distinct GnRH receptor subtypes in a teleost, the medaka *Oryzias latipes* . Endocrinology 142: 4729–4739. 11606438 10.1210/endo.142.11.8475

[42] Kusakabe R, Kusakabe T, Suzuki N (1999) In vivo analysis of two striated muscle actin promoters reveals combinations of multiple regulatory modules required for skeletal and cardiac muscle-specific gene expression. Int J Dev Biol 43: 541–554. 10610027

[43] Kusakabe T, Suzuki N (2000) Photoreceptors and olfactory cells express the same retinal gyanylyl cyclase isofom in medaka: visualization by promoter transgenics. FEBS Lett 483: 143–148. 11042270 10.1016/s0014-5793(00)02109-8

[44] Okubo K, Sakai F, Lau EL, Yoshizaki G, Takeuchi Y, et al (2006) Forebrain gonadotropin-releasing hormone neuronal development: insights from transgenic medaka and the relevance to X-linked Kallmann syndrome. Endocrinology 147: 1076–1084. 16293668 10.1210/en.2005-0468

[45] Higashijima S, Masino MA, Mandel G, Fetcho JR (2004) Engrailed-1 expression marks a primitive class of inhibitory spinal interneuron. J Neurosci 24: 5827–5839. 15215305 10.1523/JNEUROSCI.5342-03.2004 PMC6729218

[46] Li WC, Higashijima S, Parry DM, Roberts A, Soffe SR (2004) Primitive roles for inhibitory interneurons in developing frog spinal cord. J. Neurosci 24: 5840–5848. 15215306 10.1523/JNEUROSCI.1633-04.2004 PMC6729206

[47] Gosgnach S, Lanuza GM, Butt SJB, Saueressig H, Zhang Y, et al (2006) V1 spinal neurons regulate the speed of vertebrate locomotor outputs. Nature 440: 215–219. 16525473 10.1038/nature04545

[48] Okubo K, Ishii S, Ishida J, Mitani H, Naruse K, et al (2003) A novel third gonadotropin-releasing hormone receptor in the medaka *Oryzias latipes*: evolutionary and functional implications. Gene 314: 121–131. 14527724 10.1016/s0378-1119(03)00711-x

[49] Millar RP, Lu ZL, Pawson AJ, Flanagan CA, Morgan K, Maudsley SR (2004) Gonadotropin-releasing hormone receptors. Endocr Rev 25: 235–275. 15082521 10.1210/er.2003-0002

[50] Fernald RD (2009) Gonadotropin-releasing hormone receptors: Where did they come from? Endocrinology 150: 2507–2508. 19458247 10.1210/en.2009-0475 PMC5393299

[51] Okada T, Yamamoto M (1999) Differentiation of the gonad rudiment into ovary and testis in the solitary ascidian, *Ciona intestinalis* . Dev Growth Differ 41: 759–768. 10646806 10.1046/j.1440-169x.1999.00471.x

[52] Chiba S, Sasaki A, Nakayama A, Takamura K, Satoh N (2004) Development of *Ciona intestinalis* juveniles (through 2nd ascidian stage). Zool Sci 21: 285–298. 15056923 10.2108/zsj.21.285

[53] Horie T, Shinki R, Ogura Y, Kusakabe TG, Satoh N, et al (2011) Ependymal cells of chordate larvae are stem-like cells that form the adult nervous system. Nature 469: 525–528. 21196932 10.1038/nature09631

[54] Cloney RA (1982) Ascidian larvae and the events of metamorphosis. Am Zool 22: 817–826.

[55] Nakayama-Ishimura A, Chambon JP, Horie T, Satoh N, Sasakura Y (2009) Delineating metamorphic pathways in the ascidian *Ciona intestinalis* . Dev Biol 326: 357–367. 19100250 10.1016/j.ydbio.2008.11.026

[56] Inada K, Horie T, Kusakabe T, Tsuda M (2003) Targeted knockdown of an opsin gene inhibits the swimming behaviour photoresponse of ascidian larvae. Neurosci Lett 347: 167–170. 12875912 10.1016/s0304-3940(03)00689-x

[57] Tsuda M, Sakurai D, Goda M (2003) Direct evidence for the role of pigment cells in the brain of ascidian larvae by laser ablation. J Exp Biol 206: 1409–1417. 12624175 10.1242/jeb.00235

[58] Kusakabe T, Tsuda M (2007) Photoreceptive systems in ascidians. Photochem Photobiol 83: 248–252. 16939365 10.1562/2006-07-11-IR-965

[59] Horie T, Sakurai D, Ohtsuki H, Terakita A, Shichida Y, et al (2008) Pigmented and nonpigmented ocelli in the brain vesicle of the ascidian larva. J Comp Neurol 509: 88–102. 18421706 10.1002/cne.21733

[60] Horie T, Nakagawa M, Sasakura Y, Kusakabe TG, Tsuda M (2010) Simple motor system of the ascidian larva: neuronal complex comprising putative cholinergic neurons and GABAergic/glycinergic neurons. Zool Sci 27: 181–190. 20141423 10.2108/zsj.27.181

[61] Eri R, Arnold JM, Hinman VF, Green KM, Jones MK, et al (1999) Hemps, a novel EGF-like protein, plays a central role in ascidian metamorphosis. Development 126: 5809–5818. 10572055 10.1242/dev.126.24.5809

[62] van Mier P, ten Donkelaar HJ (1989) Structural and functional properties of reticulospinal neurons in the early-swimming stage *Xenopus* embryo. J Neurosci 9: 25–37. 2913206 10.1523/JNEUROSCI.09-01-00025.1989 PMC6569989

[63] Grillner S, Parker D, el Manira A (1998) Vertebrate locomotion–a lamprey perspective. Ann N Y Acad Sci 860: 1–18. 10.1111/j.1749-6632.1998.tb09035.x 9928298

[64] Soffe SR, Roberts A, Li WC (2009) Defining the excitatory neurons that drive the locomotor rhythm in a simple vertebrate: insights into the origin of reticulospinal control. J Physiol 587: 4829–4844. 19703959 10.1113/jphysiol.2009.175208 PMC2770150

[65] Soffe SR, Clarke JDW, Roberts A (1984) Activity of commissural interneurons in the spinal cord of *Xenopus* embryos. J Neurophysiol 51: 1257–1267. 6737030 10.1152/jn.1984.51.6.1257

[66] Dale N (1985) Reciprocal inhibitorv interneurones in the *Xenopus* embryo spinal cord. J Physiol 363: 61–70. 4020706 10.1113/jphysiol.1985.sp015695 PMC1192914

[67] Buchanan JT (1999) Commissural interneurons in rhythm generation and intersegmental coupling in the lamprey spinal cord. J Neurophysiol 81: 2037–2045. 10322045 10.1152/jn.1999.81.5.2037

[68] Quinlan KA, Kiehn O (2007) Segmental, synaptic actions of commissural interneurons in the mouse spinal cord. J Neurosci 27: 6521–6530. 17567813 10.1523/JNEUROSCI.1618-07.2007 PMC6672441

[69] Imai, K. S., Satoh, N. & Satou, Y. 2002. Region specific gene expressions in the central nervous system of the ascidian embryo. Gene Expr. Patterns 2, 319–321. 10.1016/s0925-4773(02)00383-0 12617820

[70] Dufour HD, Chettouh Z, Deyts C, de Rosa R, Goridis C, et al (2006) Precraniate origin of cranial motoneurons. Proc Natl Acad Sci U S A 103: 8727–8732. 16735475 10.1073/pnas.0600805103 PMC1482646

[71] Ikuta T, Saiga H (2007) Dynamic change in the expression of developmental genes in the ascidian central nervous system: revisit to the tripartite model and the origin of the midbrain-hindbrain boundary region. Dev Biol 312: 631–643. 17996862 10.1016/j.ydbio.2007.10.005

[72] Stolfi A, Levine M (2011) Neuronal subtype specification in the spinal cord of a protovertebrate. Development 138: 995–1004. 21303852 10.1242/dev.061507

[73] Nakagawa M, Miyamoto T, Ohkuma M, Tsuda M (1999) Action spectrum for the photic response of *Ciona intestinalis* (Ascidiacea, Urochordata) larvae implicates retinal proteins. Photochem Photobiol 70: 359–362. 10483365

[74] Yamagami K (1972) Isolation of a choriolytic enzyme (hatching enzyme) of the teleost, *Oryzias latipes* . Dev Biol 29: 343–348. 4652273 10.1016/0012-1606(72)90074-7

[75] Dehal P, Satou Y, Campbell RK, Chapman J, Degnan B, et al (2002) The draft genome of *Ciona intestinalis*: insights into chordate and vertebrate origins. Science 298: 2157–2167. 12481130 10.1126/science.1080049

[76] Notredame C, Higgins D, Heringa J (2000) T-Coffee: A novel method for multiple sequence alignments. J Mol Biol 302: 205–217. 10964570 10.1006/jmbi.2000.4042

[77] Gouy M, Guindon S, Gascuel O (2010) SeaView version 4: a multiplatform graphical user interface for sequence alignment and phylogenetic tree building. Mol Biol Evol 27: 221–224. 19854763 10.1093/molbev/msp259

[78] Darriba D, Taboada GL, Doallo R, Posada D (2011) ProtTest 3: fast selection of best-fit models of protein evolution. Bioinformatics 27: 1164–1165. 21335321 10.1093/bioinformatics/btr088 PMC5215816

[79] Anisimova M, Gil M, Dufayard JF, Dessimoz C, Gascuel O (2011) Survey of branch support methods demonstrates accuracy, power, and robustness of fast likelihood-based approximation schemes. Syst Biol 60: 685–99. 21540409 10.1093/sysbio/syr041 PMC3158332

[80] Shimodaira H, Hasegawa M (1999) Multiple comparisons of loglikelihoods with applications to phylogenetic inference. Mol Biol Evol 16: 1114–1116.

[81] Kishino H, Hasegawa M (1989) Evaluation of the maximum likelihood estimate of the evolutionary tree topologies from DNA sequence data, and the branching order in Hominoidea. J Mol Evol 29: 170–179. 2509717 10.1007/BF02100115

[82] Strimmer K, Rambaut A (2002) Inferring confidence sets of possibly misspecified gene trees. Proc R Soc Lond B 269: 137–142. 10.1098/rspb.2001.1862 PMC1690879 11798428

[83] Schmidt HA, Strimmer K, Vingron M, von Haeseler A (2002) TREE-PUZZLE: maximum likelihood phylogenetic analysis using quartets and parallel computing. Bioinformatics 18: 502–504. 11934758 10.1093/bioinformatics/18.3.502

[84] Satou Y, Yamada L, Mochizuki Y, Takatori N, Kawashima T, et al (2002) A cDNA resource from the basal chordate *Ciona intestinalis* . Genesis 33: 153–154. 12203911 10.1002/gene.10119

[85] Nakashima Y, Kusakabe T, Kusakabe R, Terakita A, Shichida Y, et al (2003) Origin of the vertebrate visual cycle: genes encoding retinal photoisomerase and two putative visual cycle proteins are expressed in whole brain of a primitive chordate. J Comp Neurol 460: 180–190. 12687683 10.1002/cne.10645

[86] Yoshida R, Sakurai D, Horie T, Kawakami I, Tsuda M, et al (2004) Identification of neuron-specific promoters in *Ciona intestinalis* . Genesis 39: 130–140. 15170699 10.1002/gene.20032

[87] Corbo JC, Levine M, Zeller RW (1997) Characterization of a notochord-specific enhancer from the Brachyury promoter region of the ascidian, *Ciona intestinalis* . Development 124: 589–602. 9043074 10.1242/dev.124.3.589

[88] Thompson JD, Higgins DG, Gibson TJ (1994) CLUSTAL W: improving the sensitivity of progressive multiple sequence alignment through sequence weighting, position-specific gap penalties and weight matrix choice. Nucleic Acids Res 22: 4673–4680. 7984417 10.1093/nar/22.22.4673 PMC308517

