# Supplementary figures and images for: A Conserved Non-Reproductive GnRH System in Chordates
Source: PLoS One. 2012 Jul 27;7(7):e41955. doi: 10.1371/journal.pone.0041955 (PMC3407064; doi:10.1371/journal.pone.0041955)

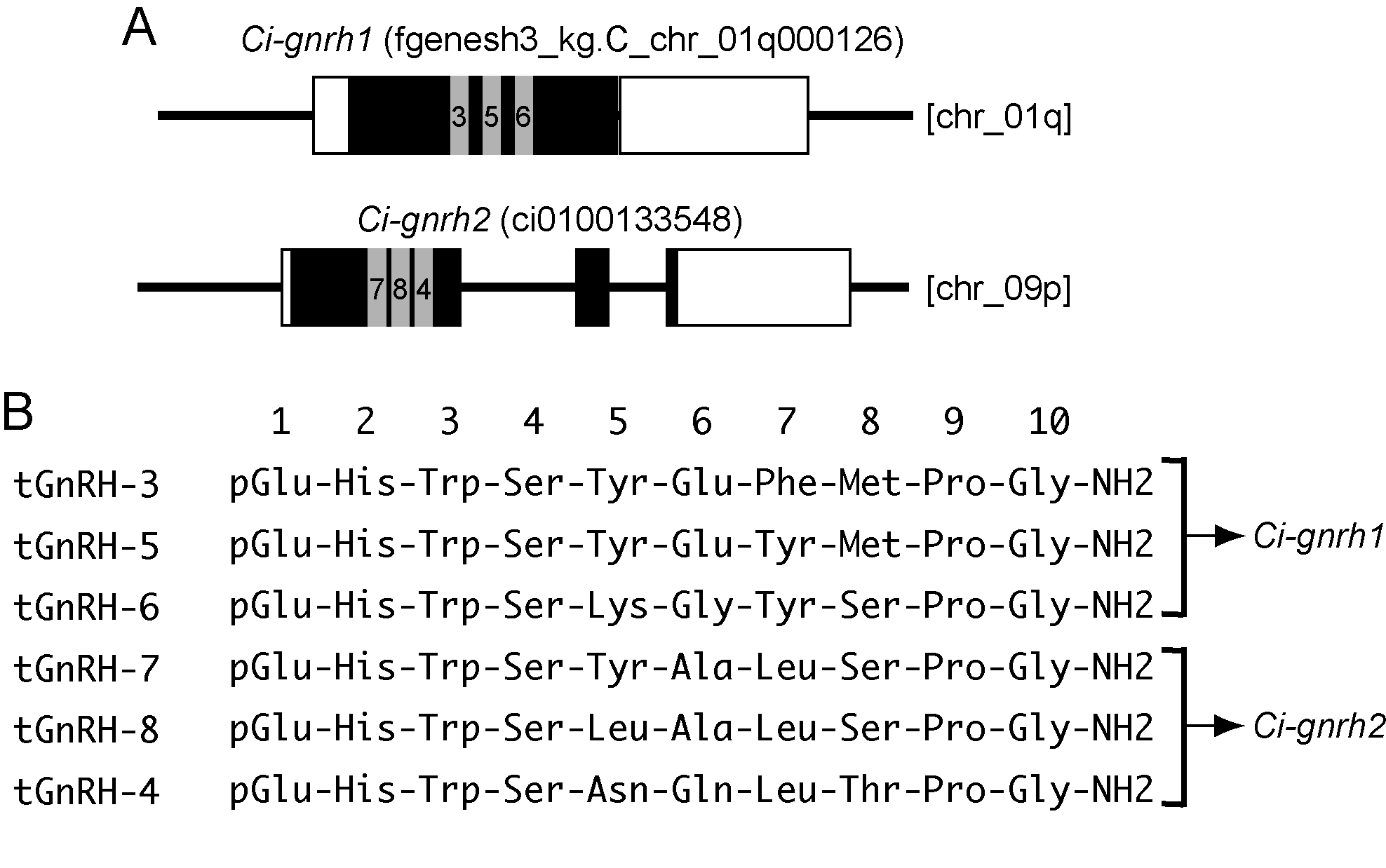

Supplement: Figure S1 — GnRH peptides and gnrh genes in Ciona intestinalis. (A) Structure of the two C. intestinalis genes encoding GnRHs. 5′ is to the left. White boxes indicate untranslated regions (noncoding exon sequences), black boxes indicate coding sequences, and each of the gray boxes with a number (3, 4, 5, 6, 7 or 8) indicates the region encoding a single GnRH peptide shown in (B). Horizontal lines indicate introns and intergenic sequences. The gene model ID for each gene is indicated in parentheses. (B) Primary structure of six C. intestinalis GnRHs. “pGlu” refers to the N-terminal pyroglutamic acid, and “Gly-NH2” indicates the C-terminal glycine amide. This figure is modified from [19]. (TIF) [file pone.0041955.s001.tif]

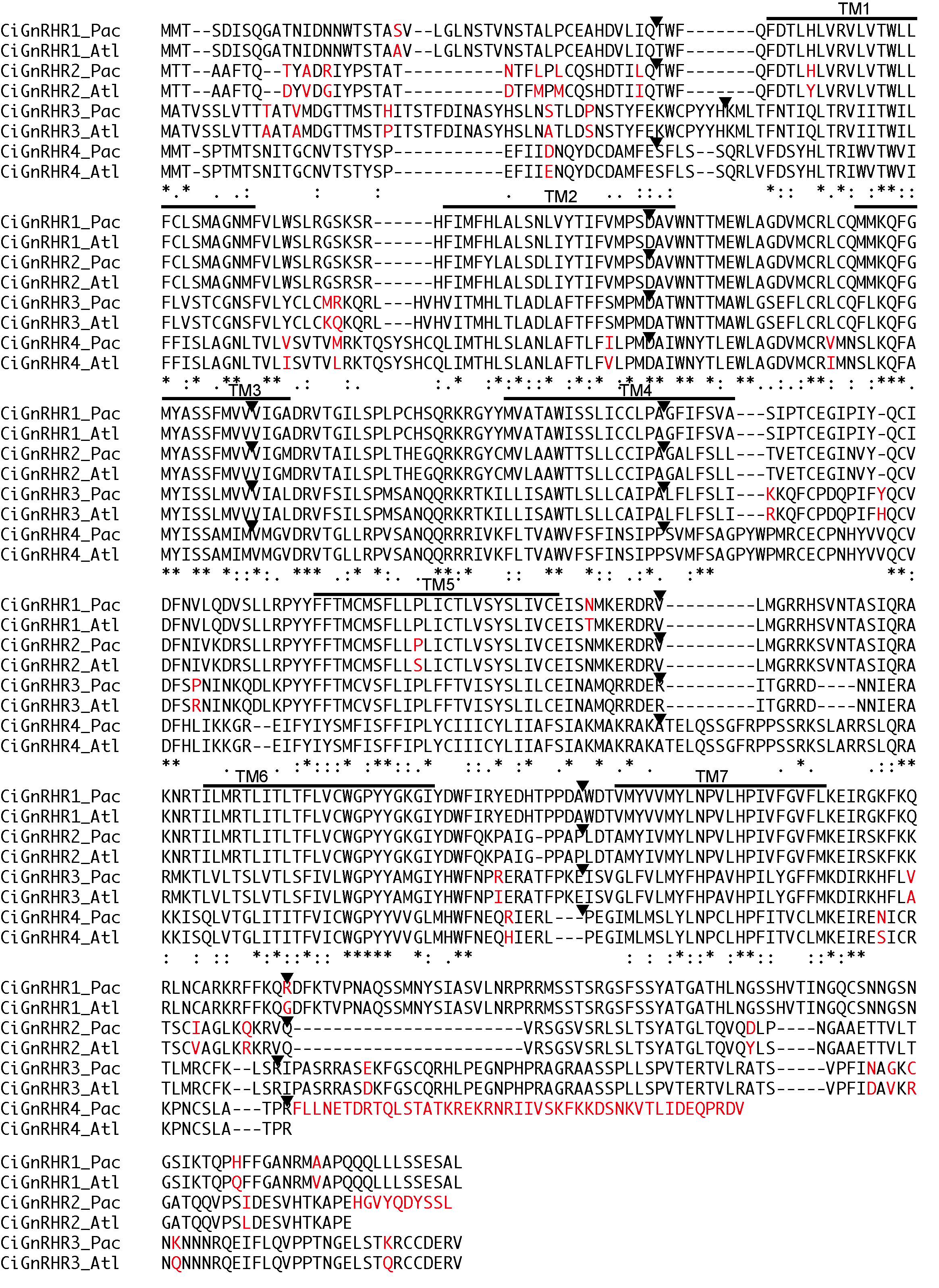

Supplement: Figure S2 — Amino acid comparison among Ci-GnRHRs. The deduced amino acid sequences of Ci-GnRHR1, Ci-GnRHR2, Ci-GnRHR3, and Ci-GnRHR4 from a Pacific population (Pac; this study) were aligned with those from an Atlantic population (Atl) [21] using the ClustalW program [88], and the alignment was optimized manually. Dashes indicate gaps introduced in the sequence to optimize the alignment. Amino acid residues showing polymorphism between Pacific and Atlantic sequences are indicated in red. The putative transmembrane domains (TM1-TM7) are indicated by horizontal lines. Triangles indicate positions of introns in the gene encoding each Ci-GnRHR. (TIF) [file pone.0041955.s002.tif]

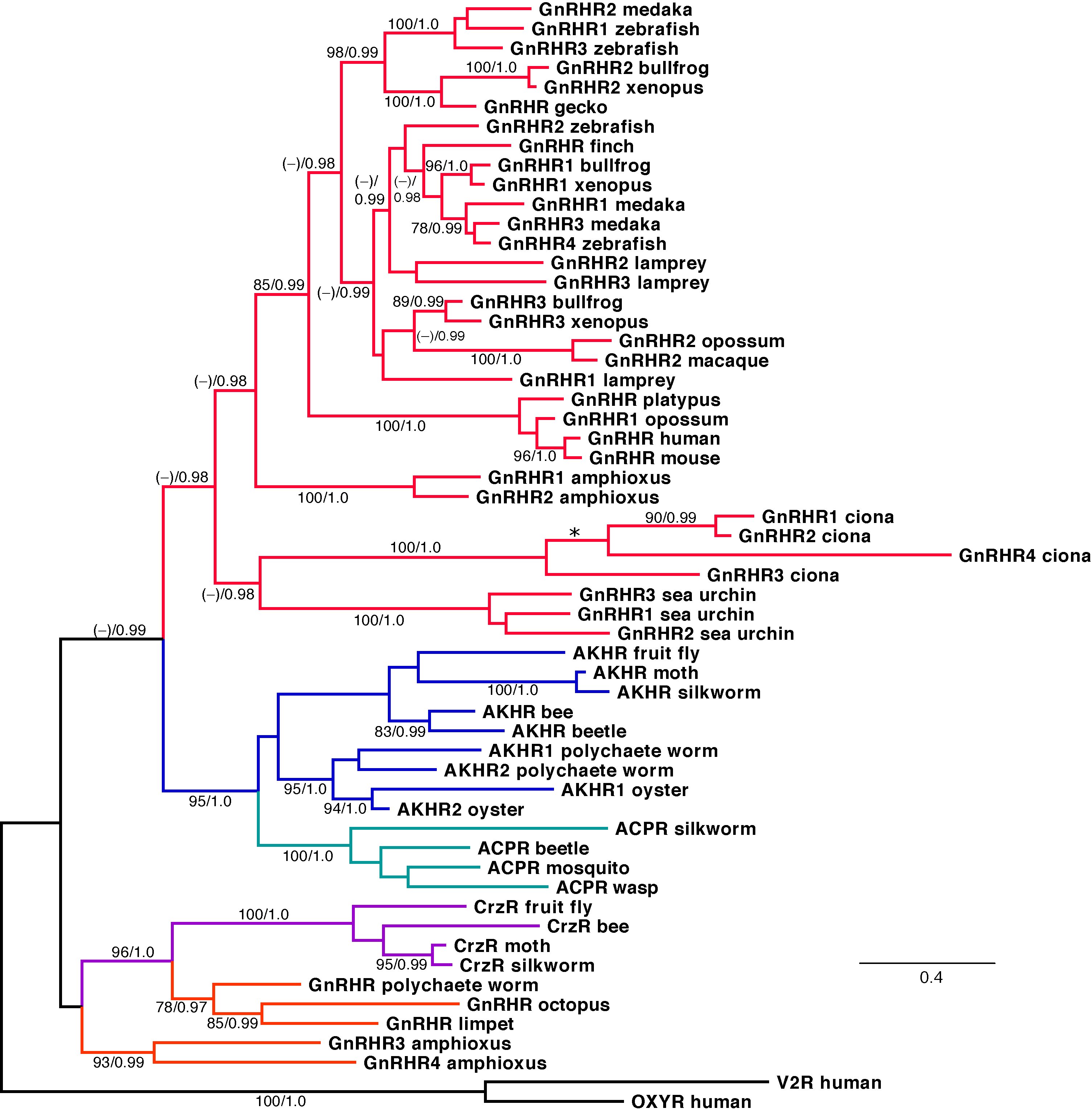

Supplement: Figure S3 — Molecular phylogeny of the GnRHR family. Maximum-likelihood (ML) trees were computed by using PhyML v3 (build 20120412), Leaphy v1.0 and RAxML v7.2.8, from a dataset comprising 57 taxa (243 sites). Human oxytocin (OXYR) and vasopressin V2 receptors (V2R) are used as outgroups. The evolutionary model was JTT+Γ4+F. Branch support values are ML non-parametric bootstrap replicates/parametric aBayes. Only bootstrap values ≥75%, and aBayes values ≥0.95 are shown. Scale bar indicates the estimated number of substitutions per site. Tree topology is from Leaphy. *It was not possible to decide which one of GnRHR3 or GnRHR4 is more closely related to the GnRHR1/GnRHR2 clade (no statistical support for either topology). (TIF) [file pone.0041955.s003.tif]

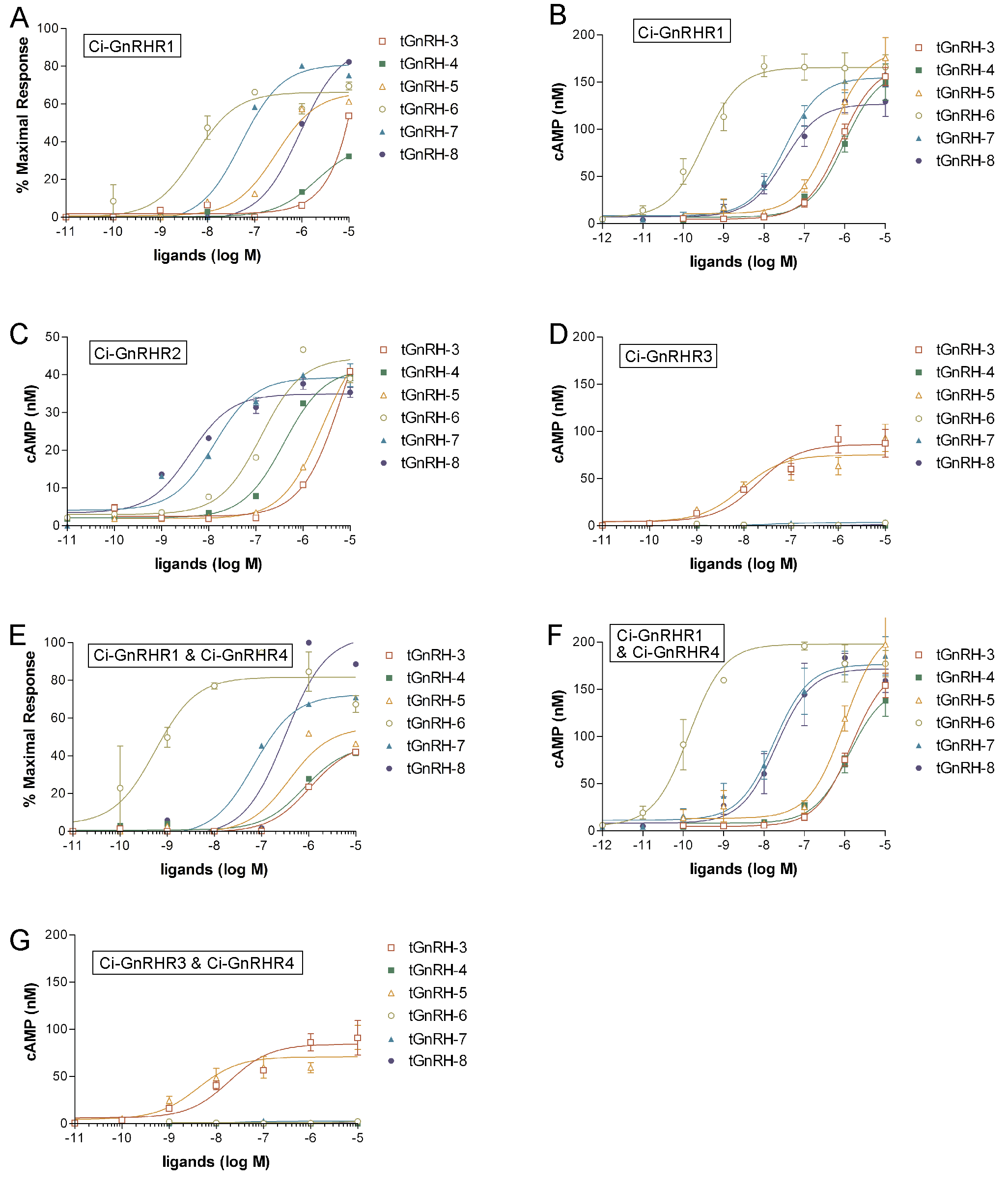

Supplement: Figure S4 — Signaling activities of various tGnRHs at Ci-GnRHRs expressed in HEK293-MSR cells. (A) Elevation of intracellular calcium ion mediated by Ci-GnRHR1 in response to tGnRHs. (B) cAMP production mediated by Ci-GnRHR1. (C) cAMP production mediated by Ci-GnRHR-2. (D) cAMP production mediated by Ci-GnRHR3. (E) Elevation of intracellular calcium ion in the cells co-expressing Ci-GnRHR1 and Ci-GnRHR4 in response to tGnRHs. (F) cAMP production in the cells co-expressing Ci-GnRHR1 and Ci-GnRHR4. (G) cAMP production in the cells co-expressing Ci-GnRHR-1 and Ci-GnRHR3. Data represent means ± S.E.M. of three independent experiments. (TIF) [file pone.0041955.s004.tif]
