# Supplementary material for: A Conserved Non-Reproductive GnRH System in Chordates
Source: PLoS One. 2012 Jul 27;7(7):e41955. doi: 10.1371/journal.pone.0041955 (PMC3407064; doi:10.1371/journal.pone.0041955)
Supplement: Table S2 — EC50 values (nM) of tGnRHs for inositol phosphate accumulation in COS-7 cells expressing Ci-GnRHR1. (DOC) [file pone.0041955.s006.doc]

Table S2. EC_50_ values (nM) of tGnRHs for inositol phosphate accumulation in COS-7 cells expressing Ci-GnRHR1.

| peptide | EC_50_ |
| --- | --- |
| tGnRH-3 | — |
| tGnRH-4 | — |
| tGnRH-5 | — |
| tGnRH-6 | 126^a^ |
| tGnRH-7 | 593^a^ |
| tGnRH-8 | 610^a^ |

^a^ Much higher concentrations of tGnRHs were required to stimulate inositol phosphate accumulation in COS-7 expressing Ci-GnRHR1 than those required for intracellular calcium ion mobilization or cAMP procduction in HEK293-MSR cells (cf. Tables 1 and 2).

A *dash* (—) denotes no response was detected.
